# First Report of TTSuV1 in Domestic Swiss Pigs

**DOI:** 10.3390/v14050870

**Published:** 2022-04-22

**Authors:** Sabrina Polster, Julia Lechmann, Julia Lienhard, Deborah Peltzer, Barbara Prähauser, Claudia Bachofen, Frauke Seehusen

**Affiliations:** 1 Institute of Veterinary Pathology, Vetsuisse Faculty, University of Zurich, 8057 Zurich, Switzerland; sabrina.polster@uzh.ch (S.P.); barbara.praehauser@uzh.ch (B.P.); 2 Institute of Virology, Vetsuisse Faculty, University of Zurich, 8057 Zurich, Switzerland; juliavictoria.lechmann@uzh.ch (J.L.); julia.lienhard@uzh.ch (J.L.); deborah.peltzer@bluewin.ch (D.P.); claudia.bachofen@uzh.ch (C.B.)

**Keywords:** TTSuV1, non-suppurative encephalitis, in situ hybridization, real-time PCR

## Abstract

Serum prevalence of Torque teno sus viruses (TTSuV1 and k2; family *Anelloviridae*) is known to be high in the porcine population worldwide but pathogenesis and associated pathomorphological lesions remain to be elucidated. In this study, quantitative real-time PCR for detection of TTSuV1 was performed in 101 porcine samples of brain tissue, with animals showing inflammatory lesions or no histological changes. Additionally, a pathomorphological and immunohistochemical characterization of possible lesions was carried out. Selected cases were screened by TTSuV1 in situ hybridization. Furthermore, TTSuV1 quantitative real-time PCR in splenic and pulmonary tissue and in situ hybridization (ISH) in spleen, lungs, mesenteric lymph node, heart, kidney, and liver were performed in 22 animals. TTSuV1 was detected by PCR not only in spleen and lung but also in brain tissue (71.3%); however, in general, spleen and lung tissue displayed lower Ct values than the brain. Positive TTSuV1 results were frequently associated with the morphological diagnosis of non-suppurative encephalitis. Single TTSuV1-positive lymphocytes were detected by ISH in the brain but also in lungs, spleen, mesenteric lymph node and in two cases of non-suppurative myocarditis. A pathogenetic role of a TTSuV1 infection as a co-factor for non-suppurative encephalitides cannot be ruled out.

## 1. Introduction

In 1997, Japanese researchers discovered a virus in the serum of a human patient (initials T.T.) with post-transfusion hepatitis [1]. This virus was identified as a non-enveloped, circular, single-stranded DNA (ssDNA) virus, which had similarities in its structure compared to other animal viruses classified within the *Circoviridae* family [2,3]. This pathogen, known as Torque teno Virus (TTV), was detected in many mammalian hosts including pigs, poultry, cattle, sheep, dogs, cats, horses and tupaias as well as in non-human primates [4,5,6,7]. TTV belongs to the family of *Anelloviridae*; in pigs, this virus is called Torque teno sus virus (TTSuV) and is subclassified into two genera, Iotatorquevirus (Torque teno sus virus 1; TTSuV1) and Kappatorquevirus (Torque teno sus virus k2; TTSuVk2). Meanwhile, the International Committee on Taxonomy of Viruses (ICTV) has approved one species for TTSuV1 (TTSuV1a) and two species for TTSuVk2 (TTSuVk2a and TTSuVk2b) [8]. TTSuVs1 and TTSuV2 have nucleotide substitution rates much higher than typical DNA viruses on a level comparable to that of RNA viruses as well as a low nucleotide identity [9,10], which leads to a wide genetic variability.

It was assumed that TTSuV1 and TTSuVk2a might interfere with the porcine host immune system and may subsequently play a role in the development of porcine circovirus-associated lymphoid lesions [11]. It is controversially discussed whether there is a direct association between TTSuV infection and the occurrence of disease. A relatively high proportion of animals with TTSuV infection are clinically healthy [12]. Furthermore, it was shown that TTSuVk2a seroprevalence is significantly higher in pigs with post-weaning multisystemic wasting syndrome (PMWS) than in unaffected healthy pigs; no such difference was detected with TTSuV1 [13,14].

In general, TTSuV seroprevalence and/or detection of viral DNA seems to be high in various regions in the Americas, Asia and Europe such as the USA, Canada, Uruguay, Brazil, China, India, Thailand, Korea, Italy and Spain [15,16,17,18,19]. In Table S1, an overview of the detection and prevalence of TTSuV in several European countries is given [15,17,20,21,22,23,24]. Epidemiological data about the occurrence of TTSuV in Switzerland were not available to date.

In human patients, a connection between TTV infection and hepatic diseases, autoimmune rheumatic diseases, hematologic disorders, acquired immunodeficiency and pulmonary diseases is suspected [25]. In a case of a 2-month-old Japanese boy with aseptic meningitis, next-generation sequencing (NGS) of cerebrospinal fluid supported evidence of TTV infection [26]. Nevertheless, this case remains speculative because no further tests were performed. In pigs with TTSuV infection, no neuropathological changes have yet been characterized so far in previous studies.

NGS has also been implemented as a diagnostic tool for identification of viruses in veterinary medicine [27]. In 2015, at the Institute of Virology, Vetsuisse Faculty Zurich, a high amount of porcine TTSuV1 was detected by NGS in the brain of a pig with suspected non-suppurative polioencephalomyelitis of viral etiology based on histopathological findings. This result raised the question whether TTSuV1 is a frequently occurring infectious agent in the Swiss pig population and may cause neurological symptoms or may also be associated with other porcine diseases. Therefore, the aim of the present study was to evaluate the correlation between TTSuV1 infection and pathomorphological changes in the brain, which may support the hypothesis that TTSuV1 is a cofactor in the pathogenesis of various diseases.

## 2. Materials and Methods

### 2.1. Animals and Tissues

The first part of this study was performed on 276 pigs from 2017 to 2019 which were sent to the Institute of Veterinary Pathology in Zurich in the course of the so-called “PathoPig” project (postmortem analysis in case of herd problems, subsidized by the Swiss government) and had undergone a full diagnostic postmortem examination. Animals from all ages were investigated, ranging from newborn suckling piglets to adult sows/boars. Tissues of brain, spleen, mesenteric lymph node, lung, heart, liver, and kidney were collected, formalin fixed and frozen at −20 °C. Subsequently, a histologic examination was conducted. In a second part of this study, 101 animals were subjected to TTSuV1 detection by quantitative real-time polymerase chain reaction (real-time PCR) and in selected cases by DNA in situ hybridization (ISH). Additionally, characterization of the brain lesions was performed using immunohistochemistry (IHC) and histochemistry. A flowchart of the experimental approach is included as supplementary material (Figure S1).

### 2.2. Histologic Examination

Central nervous system (CNS) tissue of all 276 pigs was examined in three different localizations in each case (coronary sections from frontal cortex, hippocampus, and cerebellum/brain stem) using a light microscope. Based on inflammatory status, animals were divided into three groups: animals with suppurative inflammation, animals with non-suppurative inflammation and animals without any leptomeningeal and parenchymal lesions. In addition, spleen, mesenteric lymph node, lung, heart, liver, and kidney from 22 animals were examined microscopically.

Fresh tissue was fixed in 10% buffered formalin for at least 24 h, followed by trimming and routine embedding in paraffin blocks. Afterwards, 2 µm sections were stained with hematoxylin and eosin (HE) according to standard procedures.

### 2.3. Quantitative Real-Time Polymerase Chain Reaction (Real-Time PCR) for Detection of TTSuV1

To confirm the presence of the virus in brain, spleen, and lung, a quantitative real-time PCR for TTSuV1 was performed. Selected cases, especially animals with non-suppurative inflammation of the brain, indicative of a viral infection, as well as non-altered organs were used. In total, real-time PCR was performed for samples of 101 animals: in 79 cases from brain tissue and in 22 cases from tissue of brain, spleen, and lung.

For DNA extraction, the QIAamp DNA mini kit was used (Qiagen, Basel, Switzerland). An amount of 20–25 mg of frozen organ tissue was placed into a 2 mL safe-lock tube and 180 µL of ATL buffer (Qiagen, Basel, Switzerland) and a steel bead with a diameter of 5 mm (Qiagen, Basel, Switzerland) were added. Afterwards, the tissue was homogenized for 20 s at 30 hertz frequency by the TissueLyser (Qiagen, Basel, Switzerland). The safe-lock tube was centrifuged for 3 min at full speed for the steel bead and unhomogenized organ material to build a pellet and 180 μL of the supernatant was transferred into a new 1.5 mL tube. A volume of 20 μL of proteinase K was added and incubated for 10 min at 56 °C. Afterwards, 200 μL of AL buffer (Qiagen, Basel, Switzerland) was added and the tube was vortexed. After incubating for 10 min at 70 °C, 200 μL of ethanol (96–100%) was added and the tube was vortexed for 15 s. The fluid in the tube (600 μL) was pipetted on a QIAamp Mini spin column (Qiagen, Basel, Switzerland) and further steps of extraction were carried out according to the manufacturer’s protocol.

To design primers and the probe for real-time PCR, the genome sequences of all full-length TTSuV1 available on GenBank in January 2018 were aligned and the PrimerExpress software (ThermoFisher Scientific, Waltham, MA, USA) was used with default settings. The resulting primers for TTSuV1 detection were: forward primer: nucleotide (nt) position 304–324 (5′-TCCGAATGGCTGAGTTTATGC-3′), reverse primer: nt position 361–344 (5′-CCGCCCAGTCGCTAGACA-3′) and probe: nt position 327–342 (5′-FAM-CCAGCGGTAGACAGAA-NFQ-MGB-3′). The nt positions are relative to the reference strain for TTSuV1 from GenBank (accession number NC_014070.1) and are part of the 5′-UTR, a highly conserved region of the TTSuV genome [28]. The mix for TTSuV1 PCR consisted of 10 μL of TaqMan Universal PCR master mix (Applied Biosystems, Waltham, MA, USA), 5 μL of nuclease-free water, 1 μL of the forward and reverse primers (10 μM, final concentration 0.5 μM) and the probe for TTSuV1 (5 μM, final concentration 250 nM), which resulted in a total volume of 18 μL in each well of an ABI 96-well PCR plate. Next, 2 μL of extracted DNA was pipetted in each well and mixed by pipetting up and down. To control for successful DNA extraction, the porcine mitochondrial 12S RNA-coding gene was measured using the same PCR concentrations and cycle conditions as for TTSuV1 and the forward primer p12S_F (5′-CCACCTAGAGGAGCCTGTTCTATAA-3′, nt position 541–565), the reverse primer p12S_R (5′-GGCGGTATATAGGCTGAATTGG-3′, nt position 620–599) and the probe p12S_P (5′-FAM-CGATAAACCCCGATAGACCTTACCAACCC-TAMRA-3′, nucleotide position 567–595). Positions are relative to the reference AB292606.1. On each plate, a positive extraction control (consisting of a sample known to be TTSuV1 positive) and a negative extraction control (adding 2 μL of nuclease-free water to the mix instead of DNA) were tested. The reactions were subjected to 45 cycles, run on a QuantStudio7 real-time PCR machine with the following cycling conditions: (1) 2 min at 50 °C (activation), (2) 10 min at 95 °C (activation), (3) 15 s at 95 °C (denaturation), and (4) 1 min at 60 °C (annealing, extension).

In a separate study, 23 positive brain samples with a CT value lower than 30 were used for sequencing and phylogenetic analysis. DNASTAR^®^ Lasergene 14 Software (DNASTAR, Madison, WI, USA) was used to remove primer-binding sites from the original amplicon length of 630 bp, which resulted in sequences of 592 bp in length. Phylogenetic analysis was carried out using Clone Manager (version 9) software (Sci Ed Software, Westminster, CO, USA) and running a multiway analysis. Two to three reference sequences for each species from GenBank were added to the alignment. The sequences were used in the FASTA format.

### 2.4. DNA In Situ Hybridization (ISH) for TTSuV1

Selected cases, tested for viral DNA by real-time PCR, were used for ISH—16 cases with non-suppurative inflammation of the brain (8 animals with a Ct (threshold cycle) value < 30, 4 animals with a Ct value > 30 and 4 animals with negative results in PCR of brain tissue). Furthermore, in 22 cases, regardless of the PCR result and histologic changes, TTSuV1 ISH was performed on tissues of brain, spleen, mesenteric lymph node, lung, heart, liver and kidney according to the manufacturer′s protocol (Creative Bioarray, New York, NY, USA). The results of ISH were then divided into 4 grades according to Lee et al. [11,29]: grade 0: no positive cells; grade 1: a small number of positive cells (less than 25% of the entire tissue section); grade 2: a moderate number of positive cells (25% to 50% of the entire tissue section); grade 3: a large number of positive cells (more than 50% of the entire tissue section).

The TTSuV1 DNA probe for ISH was custom designed and digoxigenin (DIG) labeled (Creative Bioarray, New York, NY, USA). The probe was targeting the 56–646 nt region of the TTSuV1 5′-UTR (NC_014070.1).

Paraffin-embedded tissue sections of 4 µm in thickness were dewaxed with xylene and rehydrated through a descending order of ethanol solutions from 100% to 70%. After washing with phosphate-buffered saline (PBS), the slides were incubated in 100 °C distilled water for 15 min followed by incubation with proteinase K solution for 8 min. The slides were washed again with PBS and then dehydrated through an increasing order of ethanol solution from 70% to 100%. For denaturation, the slides were put in a denaturing solution (Formamide, SSC 20X, 0.5M EDTA, pH 7, H20) for 5 min at 88 °C and subsequently washed with PBS. Then, the probe was diluted with hybridization buffer (1:10; Creative Bioarray, New York, NY, USA), denatured at 85 °C for 5 min and kept at 37 °C for 2 min. For hybridization, the denatured probe was applied on the slides and kept in a moist chamber at 37–42 °C overnight (HybEZTM Oven, ACD Advanced Cell Diagnostics, Newark, CA, USA). After washing the slides with PBS, post-hybridization was performed with 3% bovine serum albumin (BSA) solution for 60 min at 37 °C (HybEZTM Oven, ACD Advanced Cell Diagnostics, Newark, CA, USA). Then, the DIG-labeled hybrids were detected by anti-digoxigenin alkaline phosphatase (AP) Fab fragment (Roche Diagnostics GmbH, Mannheim, Germany) at 1:200 dilution in 3% BSA solution for 60 min at 37 °C (HybEZTM Oven, ACD Advanced Cell Diagnostics, Newark, CA, USA). Thereafter, the slides were washed with PBS and exposed to 5-bromo-4-chloro-3-indolyl-phosphate (BCIP)/nitro blue tetrazolium (NBT) substrate (Vector Laboratories, Burlingame, CA, USA) for 30 min to get an indigo reaction and finally cover slipped.

### 2.5. Immunohistochemistry (IHC) and Histochemistry

After being tested by TTSuV1 real-time PCR and ISH, morphological lesions in 8 animals with mild non-suppurative inflammation in brain sections were further characterized using immunohistochemical/histochemical staining according to standard procedures [30]. The following antibodies were used (Table 1): glial fibrillary acidic protein (GFAP) for astrocytes, ionized calcium-binding adaptor protein-1 (Iba-1) for macrophages/microglial cells, beta-amyloid precursor protein (beta-APP) for axonal damage, cluster of differentiation 3 (CD3) for T cells, cluster of differentiation 79a (CD79a) for B cells and forkhead box P3 (FOXP3) for regulatory T cells. In addition, Luxol fast blue (LFB) stain for detection of demyelination was performed according to standard procedures.

### 2.6. Statistical Analysis

Pearson′s chi square test was used for testing relationships between categorical variables (TTSuV1 positivity and certain pathomorphological findings as well as age categories and sex) with the IBM SPSS Statistics software (version 27; IBM, Armonk, NY, USA). A Pearson′s chi square value larger than the minimum expected frequency (*p* value of <0.05) indicated a statistical relationship between the categorical variables. Additionally, Student′s *t* test was performed to detect statistically significant differences of the Ct values of non-suppurative encephalitis compared to non-lesioned brains as well as in different age groups.

## 3. Results

### 3.1. Histology and TTSuV1 Detection by Real-Time PCR

In a preliminary study, brain tissue of 276 animals of different age groups was examined to gain knowledge about the incidence of inflammatory changes in the porcine brain. A total of 17 animals showed suppurative inflammation consisting of a mild to severe neutrophilic infiltration of meninges and/or parenchyma, 60 animals showed non-suppurative inflammation consisting of a mild multifocal lympho-histiocytic infiltration of meninges, neuropil and/or stroma of plexus choroideus, and 199 animals showed no changes in the neuroparenchyma and/or meninges of the brain.

In a next step, quantitative real-time PCR of brain tissue was performed in 20 suckling piglets, 32 weaning pigs, 39 fattening pigs and 10 sows (in total 101 animals); 72 animals were TTSuV1 positive (71.3%; 31 animals with a Ct value < 30, 41 animals with a Ct value > 30) and 29 animals were TTSuV1 negative (28.7%). A total of 3 animals showed suppurative inflammation, 57 animals showed non-suppurative inflammation and 41 animals showed no histological changes of the brain.

In TTSuV1-positive brains, one animal showed suppurative inflammation, 47 animals showed non-suppurative inflammation and 24 animals showed no histological alterations. To be more precise, 47 animals (82.5%) with non-suppurative inflammation were TTSuV1 positive and 10 animals (17.5%) were negative, whilst 24 animals with unremarkable histology of the brain showed TTSuV1-positive PCR results in brain tissue (58.5%) and 17 animals showed TTSuV1-negative PCR results (41.5%). Despite a more frequent appearance of TTSuV1 in brains with non-suppurative inflammation, Pearson′s chi square test did not show a significant association between TTSuV1 infection and non-suppurative inflammation in the brain (minimum expected frequency 12.63; Pearson′s chi square value 7.974, *p* = 0.005). Furthermore, the *t* test did not show statistically significant differences concerning the Ct value between animals with non-suppurative inflammation and animals without pathomorphological changes in the brain.

**Figure 1:**
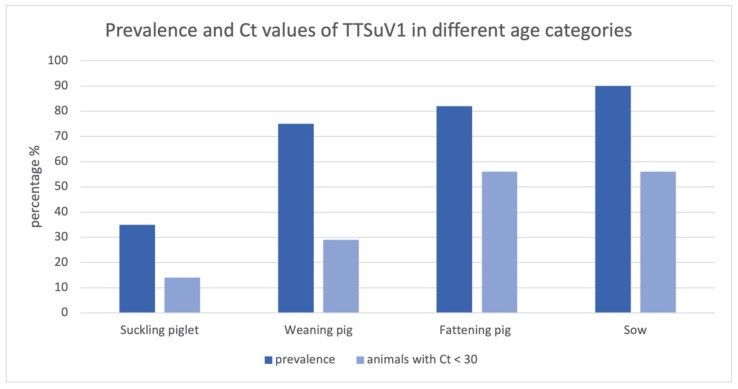
Prevalence and Ct values of TTSuV1 in different age categories; the bars show the percentages of all TTSuV1 PCR-positive animals and positive animals with a Ct < 30.

In contrast, an association could be detected between age category and TTSuV1-positive brain tissue; 7 suckling piglets out of 20 (35%), 24 weaning pigs out of 32 (75%), 32 fattening pigs out of 39 (82%) and 9 sows out of 10 (90%) had TTSuV1-positive PCR results in brain tissue (Figure 1). Pearson′s chi square test showed a significant association between TTSuV1 infection and age categories (minimum expected frequency 2.87; Pearson′s chi square value 17.000, *p* = 0.001).

Furthermore, the percentage of TTSuV1-positive animals with a Ct value lower than 30 in the brain in comparison to a higher Ct value (Ct value > 30) increases with increasing age category; 14% of all TTSuV1 PCR-positive suckling piglets, 29% of all positive weaning pigs and 56% of all positive fattening pigs as well as sows revealed Ct values < 30 (Figure 1). Statistically significant differences concerning the Ct value could be detected in suckling piglets compared to fattening pig and sows (*t* test, *p* < 0.05).

On the contrary, no association could be found between sex and TTSuV1-positive brain tissue; 70.8% of female animals and 71.7% of male animals revealed TTSuV1-positive PCR results in brain tissue.

Results of histological diagnosis, PCR and ISH results in brain tissue are summarized in Table S2.

Spleen, mesenteric lymph node, heart, liver, and kidney presented mild histologic changes only in a few cases. Lesions consisted mainly of mild lymphoid depletion, neutrophilic infiltration and follicular hyperplasia in spleen and mesenteric lymph node, focal non-suppurative myocarditis, chronic interstitial nephritis, suppurative or non-suppurative hepatitis as well as necrotic changes and fatty degeneration in the liver as well as catarrhal to suppurative bronchopneumonia or bronchiolo-interstitial pneumonia as well as atelectasis.

In addition, real-time PCR of lung tissue (*n* = 22) yielded 17 animals with positive TTSuV1 results (12 animals with a Ct value < 30, 5 animals with a Ct value > 30), and 5 animals with negative TTSuV1 results. Real-time PCR of spleen tissue (*n* = 22) revealed 17 TTSuV1-positive animals (13 animals with a Ct value < 30, 4 animals with a Ct value > 30) and 5 TTSuV1-negative animals. A total of 15 of the TTSuV1-positive animals were double-positive in spleen and lung, whereas 2 TTSuV1-positive animals were positive only in lung or spleen, respectively. However, Pearson′s chi square test showed no association between pulmonary and splenic histologic changes and TTSuV1 PCR results in tissues of spleen and lung could be detected.

Gastrointestinal symptoms followed by wasting and movement apparatus problems as well as septicemia and serosal changes were the most frequently diagnosed disease complexes from PCR tested animals (with negative and positive results) during postmortem examination. To be more specific, the animals exhibited diseases such as coli diarrhea (*n* = 15), rotavirus diarrhea with villous atrophy and fusion (*n* = 12), intestinal spirochaetosis (*n* = 8), swine dysentery (*n* = 1), torsio intestinalis (*n* = 4 animals), porcine proliferative enteropathy (*Lawsonia intracellularis* infection; *n* = 4), necrotizing enteritis of suckling piglets (*Clostridium perfringens* type C; *n* = 1), coccidiosis (*n* = 3), PMWS (post-weaning multisystemic wasting syndrome; *n* = 1), arthritis/polyarthritis (*n* = 16 animals; including *Mycoplasma hyosynoviae*, *Mycoplasma hyorhinis* and *Streptococcus suis* infection, all *n* = 1), polyserositis (*n* = 19; including Glaesser′s disease *n* = 3), septicemia (*n* = 14; including erysipelas/*n* = 1 and colisepticemia/*n* = 1), respiratory disorders (*n* = 12; including swine influenza virus infection/*n* = 1 and Bordetella bronchiseptica/*n* = 2) and edema disease (*n* = 3). No specific disease could be linked to the TTSuV1 PCR results due to the small sample size of animals for each diagnosed disease.

The results of the phylogenetic analysis are depicted in Figure S2. The sequences were also compared to publicly available sequences [10,31]. They shared 96 to 99% homology with TTSuV1 strains from different countries.

### 3.2. TTSuV1 Detection by DNA ISH and Immunohistochemical/Histochemical Investigation of the Porcine Brain

**Figure 2:**
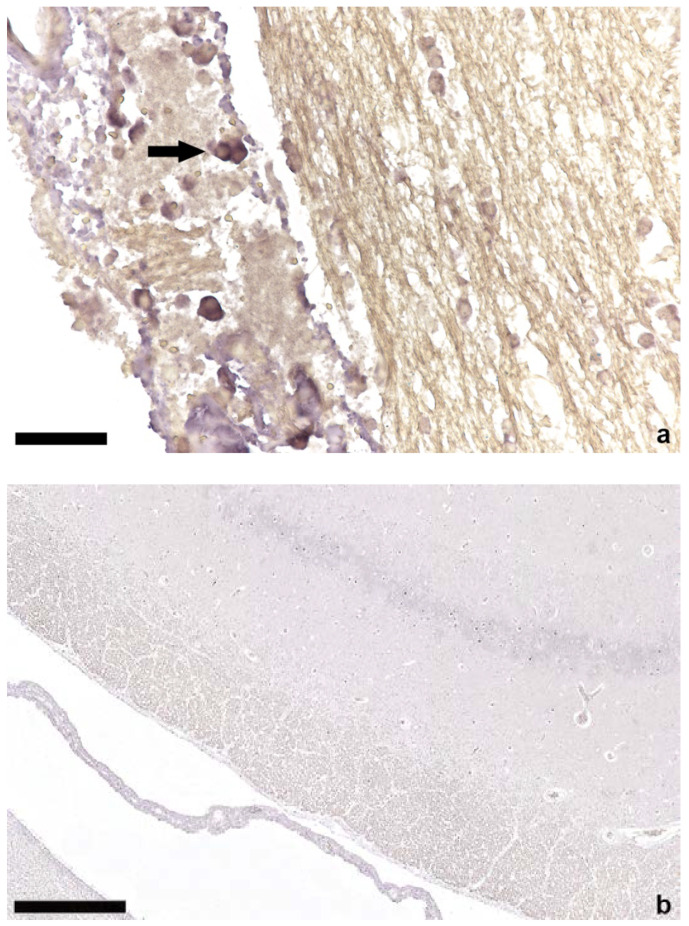
In situ hybridization of brain tissue; (**a**) positive lymphocytes (arrow) in the plexus choroideus of a TTSuV1 PCR-positive animal, bar = 50 µm; (**b**) brain parenchyma without ISH signals of a TTSuV1 PCR-negative animal, bar = 250 µm.

**Figure 3:**
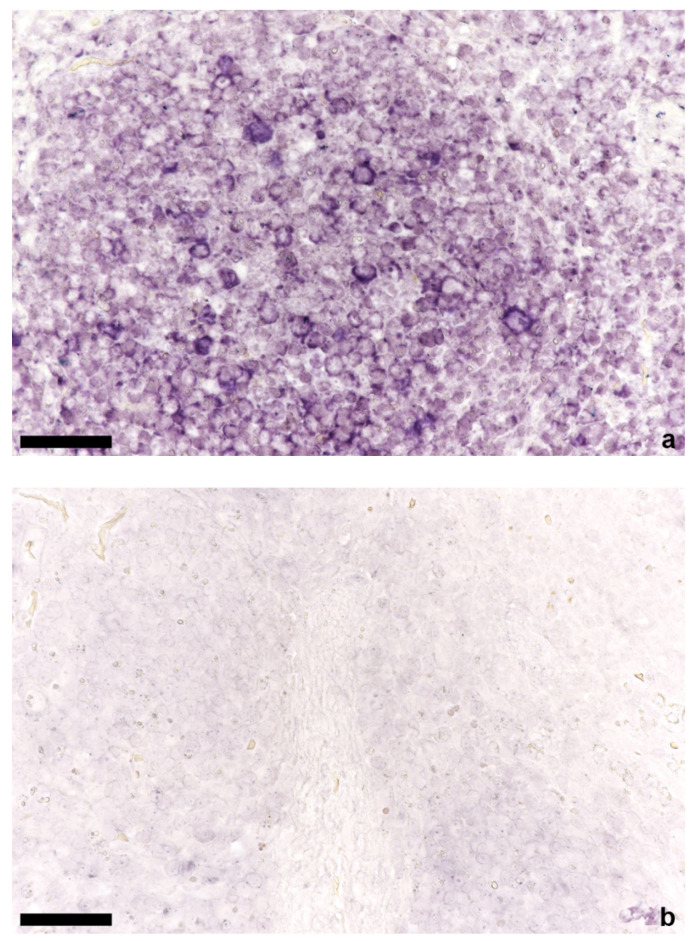
In situ hybridization of mesenteric lymph node; (**a**) positive lymphocytes in the follicle of a TTSuV1 PCR-positive animal, bar = 50 µm; (**b**) mesenteric lymph node without signals of a TTSuV1 PCR-negative animal, bar = 50 µm.

**Figure 4:**
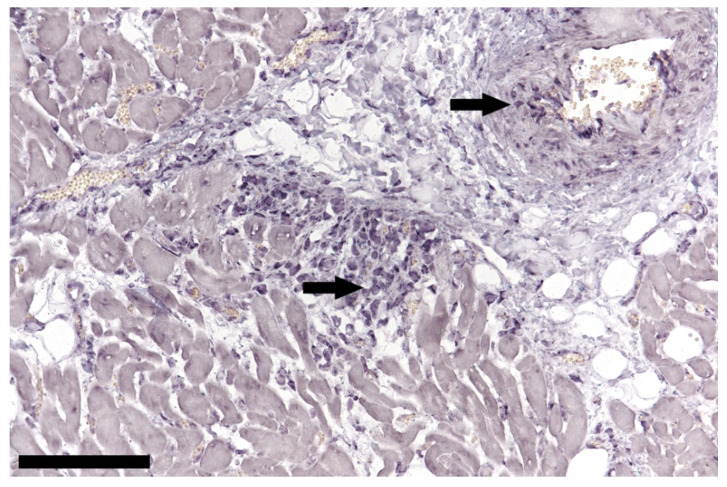
In situ hybridization of cardiac tissue; positive lymphocytes (arrows) in a focal non-suppurative myocarditis and arterial wall of a TTSuV1 PCR-positive animal, bar = 100 µm.

In 16 cases, DNA ISH was performed only in brain tissue (12 × TTSuV1 PCR positive, 4 × TTSuV1 PCR negative) and in 22 cases in tissues of brain (16 × TTSuV1 PCR positive, 6 × TTSuV1 PCR negative), spleen (17 × TTSuV1 PCR positive, 5 × TTSuV1 PCR negative), mesenteric lymph node, lung (17 × TTSuV1 PCR positive, 5 × TTSuV1 PCR negative), heart, liver, and kidney. Nucleic acids of TTSuV1 were detected in brain (Figure 2), mesenteric lymph node (Figure 3), spleen, lung, and heart (Figure 4). No TTSuV1-positive signals were present in liver and kidney (Table 2). Positive signals in brain tissue could only be found in single lymphocytes located in the plexus choroideus or sometimes in the lumen of blood vessels (grade 1 according to Lee et al.; [11,24]). More TTSuV1-positive signals (grade 1 and grade 2) were detected in mesenteric lymph node, spleen, and lung; positive lymphocytes were present in the follicle of the mesenteric lymph node, in the white pulp of the spleen as well as in the peribronchial lymphatic tissue, and the interstitium of the lung. In two cases, TTSuV1-positive signals were detected in lymphocytes of a focal non-suppurative myocarditis.

In general, animals which were TTSuV1 negative in PCR did not show positive ISH signals. One case which showed negative results in TTSuV1 PCR in the spleen and positive PCR results in brain and lung, ISH-positive cells could be detected in the splenic parenchyma as well as in mesenteric lymph node and lung (Table 3).

The immunohistochemical examination of brain sections revealed a physiological quantity and distribution of GFAP-positive astrocytes without signs of astrogliosis. The Luxol fast blue stain showed physiological myelin content in the white matter without signs of demyelination.

Beta-APP immunohistochemistry revealed mild signs of acute axonal damage as single dot-like positive signals in three cases (all TTSuV1 PCR positive). Iba1 IHC for macrophages/microglial cells showed a mild to moderate expression in all eight cases (six × TTSuV1 PCR positive, two × TTSuV1 PCR negative). In addition, four animals revealed a small amount of CD3- (but not CD79a-) positive cells in brain sections which were positive for TTSuV1 in real-time PCR; two of these four animals also presented a positive result in TTSuV1 ISH. Another animal showed CD3- and CD79a-positive signals in the brain with a positive result in TTSuV1 PCR and ISH. These findings implicate that TTSuV1-positive lymphocytes seen in ISH are most likely T lymphocytes. Regulatory T cells could not be detected in FOXP3 IHC.

## 4. Discussion

In this study, we investigated the presence of TTSuV1 in different organs with emphasis on the brain in the Swiss pig population. We showed that the prevalence of TTSuV1 infection increased with age, indicating that the older the animals get, the more likely they become TTSuV1 positive and show lower Ct values, which is consistent with the findings of Aramouni et al. [32]. The fecal–oral route is assumed to be the main route of infection for anelloviruses, but they are also transmitted by vertical and transplacental/intra-uterine route [33,34]. A Brazilian study showed similar results, suggesting that TTSuV detected in fecal samples spread to pigs of all production stages and that the viral infection rate increased with the age of the animals [35]. Similar to Blois et al. [15], we could not find a difference between males and females concerning TTSuV1 prevalence. The clinical relevance of these findings is uncertain because it is known from the literature that TTSuV represents a frequently detected viral agent in healthy and diseased pigs. Therefore, it was described that a relatively high proportion of animals with TTSuV infection are clinically healthy [12,16]. In general, TTSuV seroprevalence in pigs seems to be high (33% to 100%) in various countries such as in the USA, Thailand, Canada, China, Korea, and Spain [17]. TTSuV1 and TTSuVk2 were detected by real-time qPCR in sera of healthy pigs in Italy, with a high prevalence of 83.2%. Furthermore, co-infections were significantly more frequent than infections with single species [15]. In our study, TTSuV1 was detected by real-time PCR in several organs including spleen, lungs, and brain. Therefore, it seems that the prevalence of TTSuV1 infection in the Swiss pig population is also high.

It has also been shown that TTSuVk2a seroprevalence is significantly higher in pigs with post-weaning multisystemic wasting syndrome (PMWS) than in unaffected healthy pigs; no such difference was detected with TTSuV1 [13,14,36]. In contrast, an experimentally induced coinfection of TTSuV1 with porcine circovirus type 2 (PCV2) demonstrated an increase in the clinical severity of PMWS [37]. Moreover, increased detection of TTSuV1 was associated with B cell hyperplasia in the superficial inguinal lymph nodes, while the tissue viral load of TTSuVk2a was correlated with an increased number of macrophages in the same organ. In addition, lymphoid depletion and granulomatous inflammation, which are significant histopathological findings in PMWS-affected pigs, were correlated to the presence of TTSuVk2a-positive cells as determined by ISH [11]. In a study from the Lesser Antilles, high rates of TTSuV1 infection in many pigs showed co-infection with either PCV2 or porcine parvovirus (PPV) [38]. Additionally, gnotobiotic pigs with an experimental TTSuV1 infection did not develop any clinical symptoms but showed interstitial pneumonia, transient thymic atrophy, membranous glomerulonephropathy and moderate lymphohistiocytic infiltrates in the liver [39]. In our study, only mild and rather unspecific inflammatory lesions could be detected in several organs. Due to the vaccination scheme of pig herds in Switzerland, PCV2-associated diseases were only rarely detected in recent years [40]. Therefore, a correlation between PCV2 and TTSuV1 could not be detected in the present study.

Lee et al. [29] evaluated cell tropism and tissue distribution of TTSuV1 and TTSuVk2a by using ISH. In general, lymphoid tissues showed higher levels of positive signals and T lymphocytes were assumed to be the major target cell population for TTSuV. In our study, T lymphocytes also seemed to be the main cell population targeted by TTSuV1 in the brain. The possible explanation for lower Ct values in lungs and spleen would be the presence of lymphocytes in the bronchus-associated lymphoid tissue (BALT) and in the splenic white pulp, respectively. The role of lymphocytes as the main target cell population in TTSuV1 infection and distribution was also emphasized by the detection of TTSuV1-positive lymphocytes in two cases of non-suppurative myocarditis. In general, the role of TTSuV as a commonly distributed pathogen such as in the investigated Swiss pigs was also emphasized by the detection of TTSuV1 and one in several cell lineages (also one frozen cell culture from 1985) [41] which was interpreted as contamination. Different wildlife species, positive for TTSuV1 or TTSuV2ka, may play a role as reservoirs [42,43].

Similar to pigs, the distribution of TTV in humans suggests multisystemic infection, with virus detected in blood, liver, bone marrow, lung, spleen, pancreas, kidney, lymph node, skeletal muscle and thyroid gland [44,45,46]. In addition, it was assumed that TTV infection of the central nervous system leads to a locally increased expression of inflammatory mediators in humans, which could play a role in the pathogenesis of multiple sclerosis [47]. Although not statistically significant, this study showed a tendency towards an association between the prevalence of TTSuV1 and the occurrence of histopathological changes in the brain, which suggests that animals with mild non-suppurative inflammation in the brain (non-suppurative encephalitis/meningoencephalitis/plexus choroiditis) are more likely to be TTSuV1 positive than animals without any changes in the neuroparenchyma and/or meninges of the brain. We also detected TTSuV1-positive lymphocytes in mild non-suppurative encephalitis cases. The clinical relevance of this finding is unclear, as no anamnestic data of central nervous signs in the porcine patients were reported in most cases. Additionally, only one case report of a child with a suspected TTV infection was reported [26]. Because this assumption was only based on NGS findings, its relevance is still questionable. Nevertheless, in the first animal in which TTSuV1 was detected by NGS, co-infection with atypical porcine pestivirus (APPV) existed (non-published data), favoring the role of TTSuV1 as a co-factor of other virally induced inflammatory and/or degenerative lesions in the porcine central nervous system. Apart from non-suppurative inflammation, neuropathological changes in the brain of TTSuV1-positive brains were sparse as no obvious microgliosis, astrogliosis or demyelination were detected.

Not all animals which were positive in the TTSuV1 PCR in the brain showed a positive signal in ISH. This can be explained by the overall lower sensitivity of ISH compared to PCR. Furthermore, TTSuV1 showed high genetic diversity, which may cause diagnostic difficulties. In this investigation, we focused on the detection of TTSuV1 because TTSuVk2 is less frequently detectable as a mono-infection [16]. Furthermore, the genetic diversity between TTSuV1 and TTSuVk2 is so high that both genera cannot be detected in the same real-time PCR.

## 5. Conclusions

In summary, TTSuV1 seems to be widely distributed in the Swiss pig population and could be detected by real-time PCR not only in spleen and lung but also in brain tissue; however, spleen and lung tissue usually showed lower Ct values than the brain. TTSuV1-positive lymphocytes were detected by ISH in porcine brain tissue. Therefore, a pathogenetic role of a TTSuV1 infection as a co-factor for non-suppurative encephalitides cannot be ruled out. Nevertheless, according to the relatively small number of TTSuV1-positive lymphocytes detected by ISH, this virus most likely does not possess an exclusive role as an infectious agent. 

## Figures and Tables

**Table 1:** Antibodies used in immunohistochemistry.

Antibody	Company/Item Number	Dilution	Pretreatment
GFAP	Dako Z0334	1:600	pressure cooker pH 6
beta-APP	Millipore MAB348	1:800	pressure cooker pH 6
Iba-1	Wako 019-19741	1:750	pressure cooker pH 6
CD3	Dako M725401	1:15	pressure cooker pH 9
CD79a	Bio-Rad MCA2538GA	1:1000	pressure cooker pH 9
FOXP3	Invitrogen 14-5773-82	1:50	pressure cooker pH 6

**Table 2:** Results of TTSuV1 in situ hybridization in different tissues. Data are total numbers; positive animals/all animals tested.

Tissue	TTSuV1 ISH Positive
Brain	8/38
Mesenteric lymph node	9/22
Spleen	8/22
Lung	9/22
Heart	2/22
Liver	0/22
Kidney	0/22

**Table 3:** Results of TTSuV1 in situ hybridization in comparison to TTSuV1 real-time PCR; data are total numbers of animals.

	Brain PCR Pos *	Brain PCR Neg *	Spleen PCR Pos	Spleen PCR Neg	Lung PCR Pos	Lung PCR Neg
Brain ISH pos	8	0	2	0	2	0
Brain ISH neg	20	10	15	5	15	5
Lymph node pos	9	0	8	1	9	0
Lymph node neg	7	6	9	4	8	5
Spleen ISH pos	8	0	7	1	8	0
Spleen ISH neg	8	6	10	4	9	5
Lung ISH pos	9	0	8	1	9	0
Lung ISH neg	7	6	9	4	8	5
Heart ISH pos	2	0	2	0	2	0
Heart ISH neg	14	6	15	5	15	5
Liver ISH pos	0	0	0	0	0	0
Liver ISH neg	16	6	17	5	17	5
Kidney ISH pos	0	0	0	0	0	0
Kidney ISH neg	16	6	17	5	17	5

* pos = positive; neg = negative.

## Data Availability

The sequence of the first detection of TTSuV in a brain of a Swiss pig with non-suppurative encephalitis was submitted to GenBank under the accession number ON131018. Master theses of two authors (D.B. and J.L. (Julia Lienhard)) are available at the Zurich Open Repository and Archive of the University of Zurich (ZORA).

## Supplementary Materials

The following supporting information can be downloaded at: https://www.mdpi.com/article/10.3390/v14050870/s1, Figure S1: Flowchart depicting experimental set-up; Figure S2: Phylogenetic analysis of TTSuV1 positive samples with a Ct value < 30; Table S1: Detection and prevalence of TTSuV in different European countries; Table S2: Animal data including age, sex, histological diagnosis of the brain, Ct values of TTSuV1 PCR of brain, lung and spleen as well as results of the TTSuV1 in situ hybridization in the brain.;
