# Supplementary material for: First Report of TTSuV1 in Domestic Swiss Pigs
Source: Viruses. 2022 Apr 22;14(5):870. doi: 10.3390/v14050870 (PMC9146045; doi:10.3390/v14050870)
Supplement: Supplementary file 1 [file viruses-14-00870-s001.zip › Figure S1 and S2.pdf]

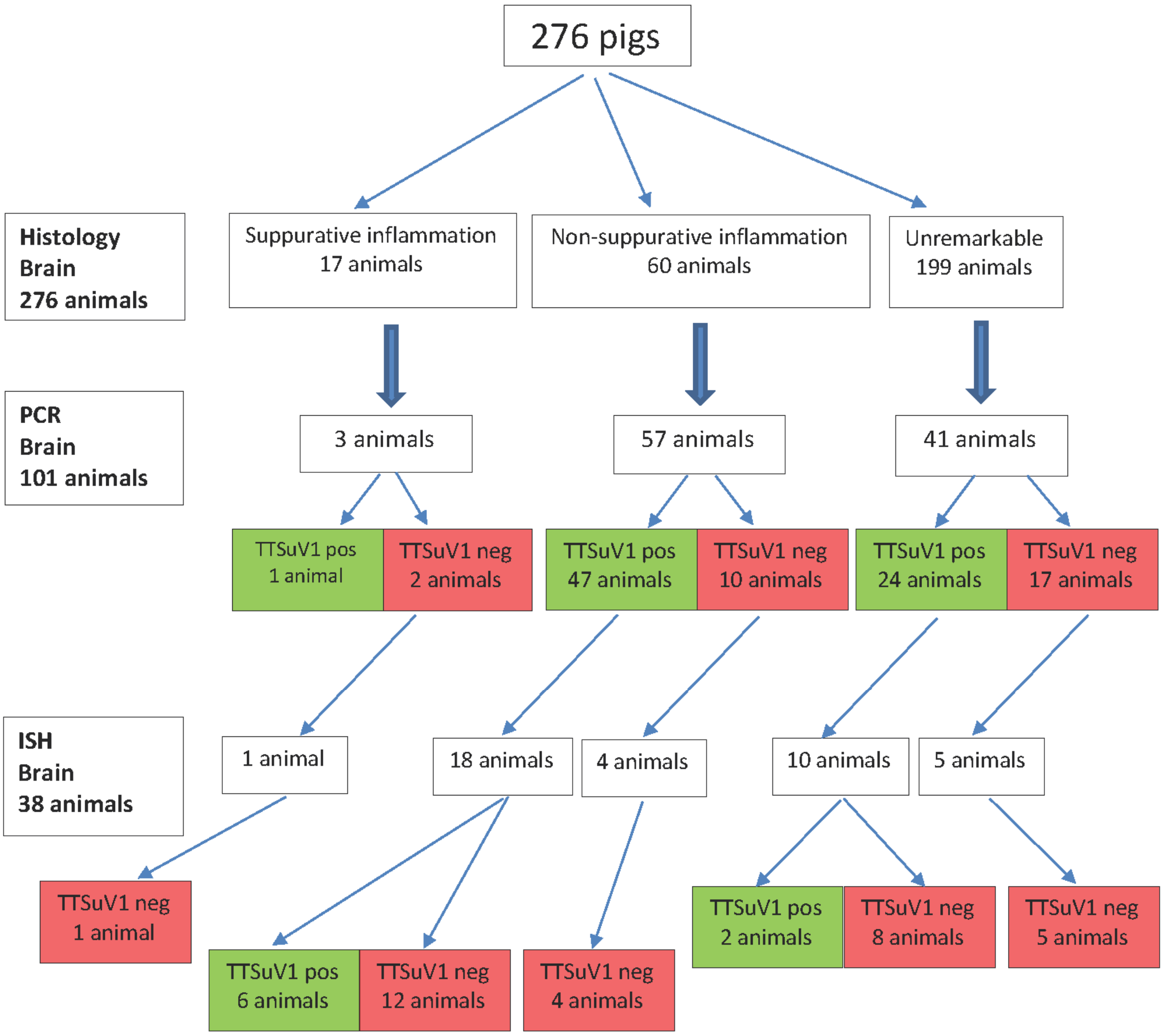

Supplementary Figure S1: Flowchart depicting experimental set-up.

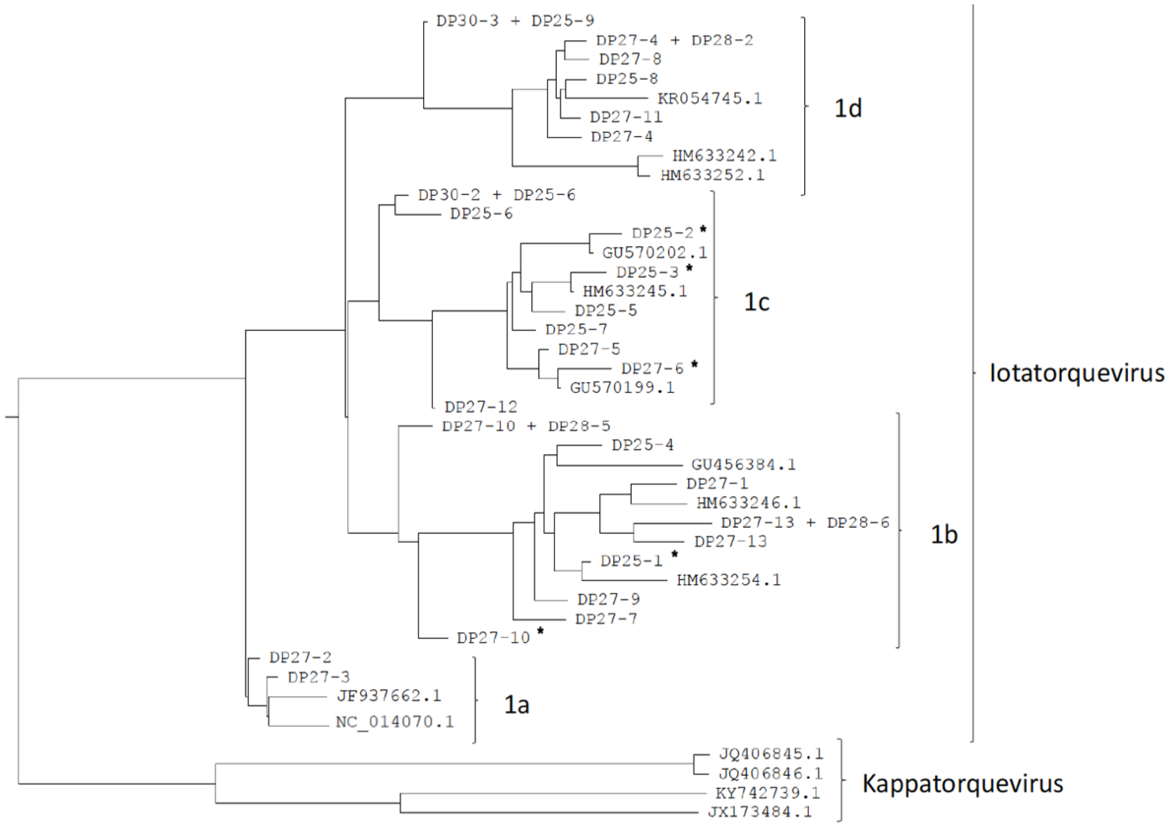

Supplemental Figure 2: Phylogenetic analysis of TTSuV1 positive samples with a Ct value < 30.

Samples with two protocol numbers (DPXX-XX + DPXX-XX) represent the two samples that were sent in for sequencing a second time due to poor or incomplete sequencing results. Two to three reference strains from GenBank were included in the analysis for each species and are named as recorded in GenBank. Asterisks mark samples of pigs with inflammatory brain lesions.

The phylogenetic analysis proved the existence of several species of TTSuV1 in Switzerland. Following the guidelines of the International Committee on Taxonomy of Viruses (ICTV), TTSuV1 only contains two species, TTSuV1a and 1b. However, this classification does not account for the very broad genetic diversity of TTSuV and is not implemented by most authors describing phylogeny. Therefore, the studies of Liu et al. (2013) and Cortey et al. (2011) were used as basis for subgrouping our sequences, thus using a classification into four species, 1a-d, because it represents better the morphology of the phylogenetic tree. The first sequencing run resulted in incomplete sequences for some of the samples. However, with one exception they were complete in the repetition. These sequences are highlighted by displaying two protocol numbers in the phylogenetic tree. For example, the sample with protocol number DP27-4 had to be sequenced a second time (re-sequencing protocol number DP28-2), but nevertheless, the sequence remained in the cluster of species TTSuV1d. From the 23 included sequences, 2 (9.1 %) belonged to the species 1a, 7 (31.8 %) to the species 1b, 8 (36.4 %) to the species 1c and 5 (22.7 %) belonged to the species 1d. One sample sequence showed a poor sequencing result with a very short sequence even after re-sequencing and was therefore not included.

The sequences of this study were also compared to all publicly available sequences using the online BLAST tool from NCBI and they were found to share 96 to 99 % homology with TTSuV1 strains from all over the world (Romania, Germany, Spain, Norway, Argentina, USA, China, South Korea and Thailand amongst others).

This work was performed as part of an master thesis (D. Peltzer). The master thesis is available at the Zurich Open Repository and Archive of the University of Zurich (ZORA).

References:
Cortey M, Pileri E, Segalés J, Kekarainen T. Globalisation and global trade influence molecular viral population genetics of Torque Teno Sus Viruses 1 and 2 in pigs. *Vet Microbiol* **2012**, *156*, 81-87.

Liu, J.; Guo, L.; Zhang, L.; Wei, Y.; Huang, L.; Wu, H.; Liu, C. Three new emerging subgroups of Torque teno sus viruses (TTSuVs) and co-infection of TTSuVs with porcine circovirus type 2 in China. *Virol J* **2013**, *10*, 189.
