# Supplementary material for: First Report of TTSuV1 in Domestic Swiss Pigs
Source: Viruses. 2022 Apr 22;14(5):870. doi: 10.3390/v14050870 (PMC9146045; doi:10.3390/v14050870)
Supplement: Supplementary file 1 [file viruses-14-00870-s001.zip › Table S1.pdf]

Supplemental Table 1: Detection and prevalence of TTSuV in different European countries

Country and year of detection	Detection method	Samples	TTSuV subtypes	Reference
Spain, 2004	PCR	Serum	porcine TTV (90%)	McKeown et al.,
Germany, 2010	Multiplex real time PCR	Serum	TTSuV1 only (20%), TTSuV2 only (49%), TTSuV1 + TTSuV2 (19%)	Gallei et al.
Czech Republic, 2011	PCR	Blood	TTSuV1 (42.9%), TTSuV2 (46.7%)	Jarosova et al.
Austria, 2011	Real time qPCR	Semen	TTSuV1, TTSuV2	Lang et al.
Croatia, 2012	PCR	Tissue of PCV2-associated lesions	TTSuV1, TTSuV2	Novosel et al.
Great Britain, 2013	Real time PCR	Serum and tissue	TTSuV1 (56% sera, 53 tissue), TTSuV2 (52% sera, 71% tissue)	McMenamy et al.
Italy, 2014	Real time qPCR	Serum	TTSuV1 (62.3%), TTSuVk2a (60.6%), TTSuVk2b (11.5%)	Blois et al.

References:

McKeown, N.E.; Fenaux, M.; Halbur, P.G.; Meng, X.J. Molecular characterization of porcine TT virus, an orphan virus, in pigs from six different countries. *Vet Microbiol* **2004**, *104*, 113-117.

Gallei, A.; Pesch, S.; Esking, W.S.; Keller, C.; Ohlinger, V.F. Porcine Torque teno virus: determination of viral genomic loads by genogroup-specific multiplex rt-PCR, detection of frequent multiple infections with genogroups 1 or 2, and establishment of viral full-length sequences. *Vet Microbiol* **2010**, *143*, 202-212.

Jarosova, V.; Pogranichniy, R.; Celer, V. Prevalence and age distribution of porcine torque teno sus virus (TTSuV) in the Czech Republic. *Folia Microbiol* **2011**, *56*, 90-94.

Lang, C.; Griessler, A.; Pirker, E.; Söllner, H.; Segalés, J.; Kekarainen, T.; Ritzmann, M. Detection of porcine Circovirus type 2 and Torque-Teno-Sus-virus 1 and 2 in semen samples of boars from an Austrian artificial insemination centre. *Tierarztl Prax Ausg G* **2011**, *39*, 201-204.

Novosel, D.; Cubric-Curik, V.; Jungic, A.; Lipej, Z. Presence of Torque teno sus virus in porcine circovirus type 2-associated disease in Croatia. *Vet Rec* **2012**, *171*, 529.

McMenamy, M.J.; McKillen, J.; McNair, I.; Duffy, C.; Blomström, A.L.; Charreyre, C.; Welsh, M.; Allan, G. Detection of a porcine boca-like virus in combination with porcine circovirus type 2 genotypes and Torque teno sus virus in pigs from postweaning multisystemic wasting syndrome (PMWS)-affected and non-PMWS-affected farms in archival samples from Great Britain. *Vet Microbiol* **2013**, *164*, 293-298.

Blois, S.; Mallus, F.; Liciardi, M.; Pilo, C.; Camboni, T.; Macera, L.; Maggi, F.; Manzin, A. High prevalence of co-infection with multiple Torque teno sus virus species in Italian pig herds. *PLoS One* **2014**, *9*, e113720.
